# Gender- and age-based differences in outcomes of mechanically ventilated ICU patients: a Chinese multicentre retrospective study

**DOI:** 10.1186/s12871-021-01555-8

**Published:** 2022-01-10

**Authors:** Jia-Gui Ma, Bo Zhu, Li Jiang, Qi Jiang, Xiu-Ming Xi

**Affiliations:** 1 Department of Critical Care Medicine, Beijing Rehabilitation Hospital, Capital Medical University, Beijing, 100144 China; 2 Department of Critical Care Medicine, Fu Xing Hospital, Capital Medical University, No.20 Fuxingmenwai Street, Xicheng District, Beijing, 100038 China; 3 Department of Critical Care Medicine, Xuan Wu Hospital, Capital Medical University, Beijing, 100053 China

**Keywords:** Gender, Age, Mechanical ventilation, Intensive care unit, Outcome, Mortality

## Abstract

**Background:**

Previous studies have suggested that the gender and/or age of a patient may influence the clinical outcomes of critically ill patients. Our aim was to determine whether there are gender- and age-based differences in clinical outcomes for mechanically ventilated patients in intensive care units (ICUs).

**Methods:**

We performed a multicentre retrospective study involving adult patients who were admitted to the ICU and received at least 24 h of mechanical ventilation (MV). The patients were divided into two groups based on gender and, subsequently, further grouped based on gender and age < or ≥ 65 years. The primary outcome measure was hospital mortality.

**Results:**

A total of 853 mechanically ventilated patients were evaluated. Of these patients, 63.2% were men and 61.5% were ≥ 65 years of age. The hospital mortality rate for men was significantly higher than that for women in the overall study population (*P* = 0.042), and this difference was most pronounced among elderly patients (age ≥ 65 years; *P* = 0.006). The durations of MV, ICU lengths of stay (LOS), and hospital LOS were significantly longer for men than for women among younger patients (*P* ≤ 0.013) but not among elderly patients. Multivariate logistic regression analysis revealed that male gender was independently associated with hospital mortality among elderly patients but not among younger patients.

**Conclusions:**

There were important gender- and age-based differences in the outcomes among mechanically ventilated ICU patients. The combination of male gender and advanced age is strongly associated with hospital mortality.

**Supplementary Information:**

The online version contains supplementary material available at 10.1186/s12871-021-01555-8.

## Introduction

Mechanical ventilation (MV) is one of the most commonly used treatment techniques in the intensive care unit (ICU). The proportion of patients receiving MV out of total ICU admissions has reached between 50 and 70% [1–5]. With our ageing population, the number of patients with MV will steadily increase, with a projected increase of 80% by 2026 when compared to 2000 [6]. Additionally, many studies have also shown that men account for more than half of the patients receiving MV in the ICU [2, 3, 7–11], and in the very elderly patients (aged ≥80 years), this proportion can reach more than 80% [12].

However, there have been few studies on the effect of gender on clinical outcomes of mechanically ventilated patients, and the results are inconsistent. Kollef et al. [13] used multivariate analysis to show that women requiring MV were at greater risk for hospital mortality than men. In contrast, two other prospective studies showed that gender was not independently associated with hospital mortality [7, 10]. It is not known whether these findings apply to other regions and patient populations. Our hypothesis was that there are significant differences in clinical outcomes between mechanically ventilated men and women, and that such gender-related differences may depend on age. To further investigate this hypothesis, we conducted a multicentre retrospective study of ICUs in Beijing.

## Materials and methods

### Study setting and design

This retrospective observational cohort study was carried out in fourteen ICUs of thirteen tertiary teaching hospitals in Beijing between January 2012 and June 2013.

Among the fourteen participating ICUs, ten were medical-surgical ICUs, two were surgical ICUs, one was a respiratory ICU, and one was a medical ICU. The number of ICU beds ranged from eight to twenty during the study period. The study was conducted in accordance with the Declaration of Helsinki (as revised in 2013). This retrospective study was approved by the Institutional Review Board of Fu Xing Hospital, Capital Medical University (Approval number: 2019FXHEC-KY167), and a waiver of informed consent was granted.

### Study population

Patients who were admitted to the ICU and received at least 24 h of invasive MV within the first 48 h of their ICU stay were eligible. Patients were excluded if they were younger than 18 years old, had incomplete datasets, were diagnosed with a neuromuscular disease, required chronic MV prior to hospital admission, or were transferred from other facilities and had already been intubated or tracheotomised. A patient was considered one case if they were admitted to the ICU several times during the study period, and only data from the first ICU admission were analysed.

In our study, older patients were defined as those ≥65 years of age at the time of hospital admission and the cohort was divided on the basis of gender and age (< or ≥ 65 years).

### Data collection

For every enrolled patient, the following data were recorded: demographic and epidemiological characteristics; the source of ICU admission; comorbidities; the severity of the illness; the primary indication for MV; arterial blood gas measurements; MV parameters and settings; the sedatives, analgesics, and neuromuscular blockers (NMBs) used during MV; the total duration of MV; the number of ventilator-free days; the occurrence of successful weaning within 30 days; the ICU and hospital lengths of stay (LOS); ICU and hospital costs; events occurring over the course of MV; the occurrence of withholding or withdrawing life-sustaining treatments; and the discharge destination. The severity of illness was assessed using the Acute Physiology and Chronic Health Evaluation (APACHE) II scoring system [14]. In this study, we adopted the acute respiratory distress syndrome (ARDS) Network predicted body weight (PBW) equation [15] to standardize mechanical ventilation tidal volumes setting.

### Outcomes of interest

The primary outcome of interest was hospital mortality. The secondary outcomes included the duration of MV, the hospital and ICU LOS, and ICU mortality.

### Statistical analysis

Statistical analyses were carried out using SPSS 21.0 (SPSS Inc., Chicago, Illinois, USA). Data are expressed as the mean ± standard deviation for normally distributed continuous variables, the median (interquartile range) for nonnormally distributed variables, and the number (percentage) for categorical variables. Continuous variables were compared using the Mann–Whitney U test. Categorical variables were compared using the chi-square test or Fisher’s exact test. Multiple logistic regression with forwards stepwise selection was used to determine the risk factors for hospital mortality. Variables with a *P* value < 0.20 during univariate analysis as well as the variable of gender were entered into the multivariate analysis. To investigate how gender-related differences might depend on age, we prospectively chose 65 years of age as the cut-off and performed two separate multivariate analyses. A two-sided *P* value < 0.05 was considered statistically significant.

## Results

### Baseline characteristics

During the study period, we evaluated 875 patients who were admitted to the ICUs and received mechanical ventilation for at least 24 h. Twenty-two patients were excluded from the study for reasons including an age < 18 years (*n* = 2), readmission to the ICU (*n* = 4), and incomplete clinical data (*n* = 16). Therefore, 853 patients were enrolled in this study. Of these, 539 (63.2%) were men and 314 (36.8%) were women. There were 328 patients under the age of 65 years (38.5%) and 525 patients aged 65 years and older (61.5%). The baseline characteristics of the study population are shown in Table 1.

**Table 1:** Baseline characteristics of mechanically ventilated ICU patients stratified by age and gender

Variables	All women (***n*** = 314)	All men (***n*** = 539)	***P*** value	Women < 65 years (***n*** = 120)	Men < 65 years (***n*** = 208)	***P*** value	Women ≥ 65 years (***n*** = 194)	Men ≥ 65 years (***n*** = 331)	***P*** value
Age (years)	72 (55, 79)	72 (55, 80)	0.546	49 (35, 58)	51 (41, 58)	0.298	78 (73, 82)	78 (73, 83)	0.356
BMI (kg/m^2^)	23 (21, 26)	23 (21, 25)	0.156	23 (21, 26)	24 (21, 26)	0.799	23 (20, 26)	23 (21, 24)	0.057
History of smoking	30 (9.6)	271 (50.3)	< 0.001	6 (5.0)	112 (53.8)	< 0.001	24 (12.4)	159 (48.0)	< 0.001
Past history of surgery	82 (26.1)	133 (24.7)	0.641	35 (29.2)	52 (25.0)	0.410	47 (24.2)	81 (24.5)	0.950
NIMV before ICU admission	36 (11.5)	66 (12.2)	0.735	14 (11.7)	27 (13.0)	0.729	22 (11.3)	39 (11.8)	0.879
Source of ICU admission									
Medical ward	63 (20.1)	122 (22.6)	0.380	15 (12.5)	28 (13.5)	0.804	48 (24.7)	94 (28.4)	0.363
Surgical ward	145 (46.2)	252 (46.8)	0.871	64 (53.3)	113 (54.3)	0.862	81 (41.8)	139 (42.0)	0.957
Emergency department	97 (30.9)	150 (27.8)	0.342	34 (28.3)	60 (28.8)	0.921	63 (32.5)	90 (27.2)	0.198
Others	9 (2.9)	15 (2.8)	0.943	7 (5.8)	7 (3.4)	0.287	2 (1.0)	8 (2.4)	0.429
APACHE II scores	15 (11, 21)	17 (11, 23)	0.227	12 (8, 18)	14 (8, 19)	0.568	18 (13, 24)	19 (13, 24)	0.376
Comorbidities									
Hypertension	156 (49.7)	247 (45.8)	0.277	28 (23.3)	49 (23.6)	0.963	128 (66.0)	198 (59.8)	0.160
Diabetes	99 (31.5)	134 (24.9)	0.035	16 (13.3)	26 (12.5)	0.828	83 (42.8)	108 (32.6)	0.020
Chronic renal failure	40 (12.7)	57 (10.6)	0.337	10 (8.3)	15 (7.2)	0.712	30 (15.5)	42 (12.7)	0.372
Chronic heart failure	32 (10.2)	48 (8.9)	0.534	2 (1.7)	7 (3.4)	0.578	30 (15.5)	41 (12.4)	0.320
COPD	29 (9.2)	56 (10.4)	0.587	3 (2.5)	5 (2.4)	1.000	26 (13.4)	51 (15.4)	0.531
Cirrhosis	1 (0.3)	10 (1.9)	0.109	1 (0.8)	6 (2.9)	0.400	0 (0.0)	4 (1.2)	0.309
Pulmonary fibrosis	6 (1.9)	19 (3.5)	0.178	4 (3.3)	6 (2.9)	1.000	2 (1.0)	13 (3.9)	0.054
Cancer	35 (11.1)	69 (12.8)	0.476	11 (9.2)	15 (7.2)	0.528	24 (12.4)	54 (16.3)	0.220
Stroke	56 (17.8)	121 (22.4)	0.109	4 (3.3)	27 (13.0)	0.004	52 (26.8)	94 (28.4)	0.694
No. of comorbidities			0.592			0.590			0.053
None	91 (29.0)	174 (32.3)		70 (58.3)	112 (53.8)		21 (10.8)	62 (18.7)	
1	88 (28.0)	147 (27.3)		31 (25.8)	54 (26.0)		57 (29.4)	93 (28.1)	
≥ 2	135 (43.0)	218 (40.4)		19 (15.8)	42 (20.2)		116 (59.8)	176 (53.2)	
Primary indication for MV									
ARDS	30 (9.6)	47 (8.7)	0.682	16 (13.3)	16 (7.7)	0.907	14 (7.2)	31 (9.4)	0.396
Postoperative	127 (40.4)	195 (36.2)	0.215	58 (48.3)	100 (48.1)	0.964	69 (35.6)	95 (28.7)	0.101
Congestive heart failure	23 (7.3)	25 (4.6)	0.101	5 (4.2)	4 (1.9)	0.397	18 (9.3)	21 (6.3)	0.216
Aspiration	4 (1.3)	15 (2.8)	0.150	0 (0.0)	3 (1.4)	0.472	4 (2.1)	12 (3.6)	0.314
Pneumonia	49 (15.6)	111 (20.6)	0.072	11 (9.2)	28 (13.5)	0.247	38 (19.6)	83 (25.1)	0.150
Sepsis	14 (4.5)	34 (6.3)	0.258	7 (5.8)	15 (7.2)	0.631	7 (3.6)	19 (5.7)	0.277
Trauma	0 (0.0)	11 (2.0)	0.026	0 (0.0)	9 (4.3)	0.050	0 (0.0)	2 (0.6)	0.533
Cardiac arrest	10 (3.2)	17 (3.2)	0.980	2 (1.7)	6 (2.9)	0.751	8 (4.1)	11 (3.3)	0.636
COPD or asthma	17 (5.4)	33 (6.1)	0.671	1 (0.8)	3 (1.4)	1.000	16 (8.2)	30 (9.1)	0.750
Other chronic pulmonary diseases	7 (2.2)	10 (1.9)	0.706	5 (4.2)	3 (1.4)	0.242	2 (1.0)	7 (2.1)	0.565
Coma	22 (7.0)	18 (3.3)	0.015	9 (7.5)	10 (4.8)	0.315	13 (6.7)	8 (2.4)	0.016
Other	11 (3.5)	23 (4.3)	0.582	6 (5.0)	11 (5.3)	0.910	5 (2.6)	12 (3.6)	0.513
Arterial blood gas analysis prior to MV									
pH	7.36 (7.25, 7.42)	7.36 (7.25, 7.42)	0.885	7.38 (7.29, 7.44)	7.36 (7.25, 7.42)	0.162	7.34 (7.23, 7.40)	7.35 (7.25, 7.42)	0.200
Pao_2_/F_I_o_2_	183 (120, 285)	168 (112, 273)	0.412	194 (117, 319)	181 (118, 314)	0.941	180 (120, 248)	160 (110, 255)	0.268
Paco_2_ (mmHg)	40 (33, 52)	40 (34, 52)	0.790	36 (32, 42)	39 (33, 47)	0.112	42 (34, 64)	41 (34, 56)	0.359
Spo_2_ (%)	95 (89, 100)	95 (88, 99)	0.032	97 (89, 100)	95 (90, 99)	0.142	95 (89, 99)	93 (88, 98)	0.100

Data are expressed as the median (interquartile range), and number (percentage). *ICU* intensive care unit, *BMI* body mass index, *NIMV* noninvasive mechanical ventilation, *APACHE II* Acute Physiology and Chronic Health Evaluation II, *COPD* chronic obstructive pulmonary disease, *MV* mechanical ventilation, *ARDS* acute respiratory distress syndrome, *Pao*_*2*_ partial pressure of oxygen in arterial blood, *F*_*I*_*o*_*2*_ fraction of inspired oxygen, *Paco*_*2*_ partial pressure of carbon dioxide in arterial blood, *Spo*_*2*_ pulse oxygen saturation.

Overall, there were no significant differences between the genders in age, body mass index (BMI), the need for noninvasive mechanical ventilation (NIMV) before ICU admission, the source of ICU admission, APACHE II scores, or the number of comorbidities. There was also no significant difference in the primary indication for MV between the genders, except for reasons due to trauma and coma. However, we found a significant difference in the proportion of men and women who had a history of smoking (50.3% vs. 9.6%, respectively; *P* < 0.001). Before the onset of MV, men tended to have a lower pulse oxygen saturation (Spo_2_; *P* = 0.032).

However, both in the overall study population and in the older age group, when we compared the APACHE II scores according to the indication for mechanical ventilation, we found that patients had significantly higher APACHE II scores, as was the case for men in the ARDS category (*P* = 0.032 and *P* = 0.036, respectively) and women in the cardiac arrest category (*P* = 0.035 and *P* = 0.026, respectively; see Additional file 1).

### Management of mechanically ventilated ICU patients

Table 2 shows the data related to mechanically ventilated patient management, which was stratified by age and gender. There was a significant difference in the tidal volumes setting between men and women (*P* < 0.001), which remained significant even after stratification by age (*P* < 0.001). The incidence rates of ventilator-associated pneumonia (VAP) and tracheostomy in both men and women were high, but the incidence rate of self-extubation was low. Among the younger age group (< 65 years), we found that the incidence rates of VAP and tracheostomy were significantly higher among men than among women. However, there was no significant difference in these rates among the older age group (≥ 65 years). No differences were found between men and women in the choice of MV mode, the use of positive end-expiratory pressure (PEEP), or the use of sedatives, analgesics and NMBs.

**Table 2:** Management of mechanical ventilation in ICU patients stratified by age and gender

Variables	All women (***n*** = 314)	All men (***n*** = 539)	***P*** value	Women < 65 years (***n*** = 120)	Men < 65 years (***n*** = 208)	***P*** value	Women ≥ 65 years (***n*** = 194)	Men ≥ 65 years (***n*** = 331)	***P*** value
Modes and parameters at the beginning of MV									
Modes of MV			0.099			0.375			0.055
VCV	76 (24.2)	99 (18.4)		32 (26.7)	39 (18.8)		44 (22.7)	60 (18.1)	
PCV	28 (8.9)	72 (13.4)		11 (9.2)	20 (9.6)		17 (8.8)	52 (15.7)	
PSV	73 (23.2)	111 (20.6)		27 (22.5)	46 (22.1)		46 (23.7)	65 (19.6)	
SIMV	19 (6.1)	27 (5.0)		5 (4.2)	15 (7.2)		14 (7.2)	12 (3.6)	
SIMV+PSV	113 (36.0)	223 (41.4)		42 (35.0)	86 (41.3)		71 (36.6)	137 (41.4)	
Others	5 (1.6)	7 (1.3)		3 (2.5)	2 (1.0)		2 (1.0)	5 (1.5)	
Parameters setting									
Tidal volumes (mL/kg PBW)	8.20 (7.38, 8.88)	6.77 (6.07, 7.45)	< 0.001	8.25 (7.38, 9.01)	6.83 (6.32, 7.59)	< 0.001	8.07 (7.38, 8.70)	6.68 (6.07, 7.37)	< 0.001
Applied PEEP (cmH_2_O)	5 (5, 8)	6 (5, 8)	0.298	5 (5, 8)	6 (5, 8)	0.617	6 (5, 8)	6 (5, 8)	0.339
Peak pressure (cmH_2_O)	24 (20, 28)	24 (20, 28)	0.604	24 (20, 27)	24 (19, 28)	0.702	24 (20, 28)	24 (20, 29)	0.350
Plateau pressure (cmH_2_O)	17 (13, 21)	18 (14, 20)	0.759	18 (14, 21)	18 (13, 20)	0.634	17 (12, 21)	18 (14, 22)	0.472
Use of sedatives	258 (82.2)	452 (83.9)	0.523	104 (86.7)	176 (84.6)	0.613	154 (79.4)	276 (83.4)	0.250
Use of analgesics	196 (62.4)	334 (62.0)	0.895	90 (75.0)	149 (71.6)	0.509	106 (54.6)	185 (55.9)	0.781
Use of NMBs	11 (3.5)	23 (4.3)	0.582	5 (4.2)	8 (3.8)	1.000	6 (3.1)	15 (4.5)	0.417
Events occurring over the course of MV									
Barotrauma ^a^	8 (2.5)	6 (1.1)	0.112	4 (3.3)	3 (1.4)	0.456	4 (2.1)	3 (0.9)	0.472
VAP	51 (16.2)	103 (19.1)	0.294	9 (7.5)	34 (16.3)	0.022	42 (21.6)	69 (20.8)	0.828
Self-extubation	5 (1.6)	5 (0.9)	0.589	1 (0.8)	1 (0.5)	1.000	4 (2.1)	4 (1.2)	0.688
Reintubation	21 (6.7)	24 (4.5)	0.159	5 (4.2)	5 (2.4)	0.575	16 (8.2)	19 (5.7)	0.266
Tracheotomy	44 (14.0)	93 (17.3)	0.214	13 (10.8)	40 (19.2)	0.047	31 (16.0)	53 (16.0)	0.992
PMV ^b^	37 (11.8)	71 (13.2)	0.556	6 (5.0)	22 (10.6)	0.082	31 (16.0)	49 (14.8)	0.717

Data are expressed as the median (interquartile range), and number (percentage). *ICU* intensive care unit, *MV* mechanical ventilation, *VCV* volume-controlled ventilation, *PCV* pressure-controlled ventilation, *SIMV* synchronized intermittent mandatory ventilation, *PSV* pressure support ventilation, *PBW* predicted body weight, *PEEP* positive end-expiratory pressure, *NMBs* neuromuscular blockers, *VAP* ventilator-associated pneumonia, *PMV* prolonged mechanical ventilation.

^a^ Barotrauma refers to the development of at least one of the following: interstitial emphysema, pneumothorax, pneumomediastinum, pneumoperitoneum or subcutaneous emphysema

^b^ PMV was defined as the need for mechanical ventilation for more than 21 days

### Clinical outcomes

As shown in Table 3, the duration of MV, ICU LOS, and hospital LOS were significantly longer for men than for women among the younger age group (*P* ≤ 0.013), but no significant differences were observed in these times among the older age group.

**Table 3:** Outcomes of mechanically ventilated ICU patients stratified by age and gender

Variables	All women (***n*** = 314)	All men (***n*** = 539)	***P*** value	Women < 65 years (***n*** = 120)	Men < 65 years (***n*** = 208)	***P*** value	Women ≥ 65 years (***n*** = 194)	Men ≥ 65 years (***n*** = 331)	***P*** value
Ventilator-free days within 30 days ^a^ (days)	23 (0, 27)	20 (0, 27)	0.025	26 (1, 28)	24 (9, 27)	0.258	19 (0, 27)	11 (0, 26)	0.024
Successful weaning within 30 days ^b^	202 (64.3)	320 (59.4)	0.151	86 (71.7)	154 (74.0)	0.641	116 (59.8)	166 (50.2)	0.032
Total duration of MV (hours)	104 (46, 236)	136 (60, 287)	0.008	73 (41, 160)	112 (58, 237)	0.003	125 (56, 339)	154 (64, 328)	0.255
ICU LOS (days)	7 (4, 15)	9 (5, 19)	0.001	5 (3, 10)	8 (4, 16)	< 0.001	9 (4, 20)	11 (5, 21)	0.173
Hospital LOS (days)	21 (12, 35)	24 (14, 40)	0.015	19 (11, 36)	25 (14, 40)	0.013	21 (13, 35)	24 (14, 40)	0.252
ICU mortality	85 (27.1)	178 (33.0)	0.069	24 (20.0)	37 (17.8)	0.620	61 (31.4)	141 (42.6)	0.011
Hospital mortality	90 (28.7)	191 (35.4)	0.042	25 (20.8)	40 (19.2)	0.726	65 (33.5)	151 (45.6)	0.006
ICU costs (10,000 CNY)	5.5 (2.7, 10.9)	7.3 (3.7, 14.7)	< 0.001	4.4 (2.1, 7.9)	6.7 (3.3, 12.5)	< 0.001	7.2 (3.5, 13.4)	7.8 (4.1, 15.5)	0.067
Hospital costs (10,000 CNY)	9.9 (5.5, 15.5)	12.0 (7.2, 19.9)	< 0.001	7.3 (4.2, 13.1)	11.9 (6.8, 20.3)	< 0.001	10.9 (5.9, 16.7)	12.1 (7.2, 19.9)	0.041
Withhold/withdraw life sustaining treatments	47 (15.0)	82 (15.2)	0.914	14 (11.7)	24 (11.5)	0.972	33 (17.0)	58 (17.6)	0.869
Discharge destination			0.010			0.030			0.290
Home	195 (87.1)	265 (76.1)		83 (87.4)	124 (73.8)		112 (86.8)	141 (78.3)	
Respiratory care ward	8 (3.6)	15 (4.3)		3 (3.2)	5 (3.0)		5 (3.9)	10 (5.6)	
Nursing home	3 (1.3)	10 (2.9)		0 (0.0)	2 (1.2)		3 (2.3)	8 (4.4)	
Other hospital	18 (8.0)	58 (16.7)		9 (9.5)	37 (22.0)		9 (7.0)	21 (11.7)	

Data are expressed as the median (interquartile range), and number (percentage). *ICU* intensive care unit, *MV* mechanical ventilation, *LOS* length of stay, *CNY* Chinese Yuan

^a^ Ventilator-free days within 30 days are defined as 30 minus the total number of days with invasive MV. Nonsurvivors were considered to have 0 ventilator-free days

^b^ Successful weaning from MV was defined as complete respiratory autonomy for at least 48 h

Despite the observation that men and women aged 65 years or older had similar severities upon ICU admission, mortality was higher among men than among women in the ICU (42.6% vs. 31.4%, respectively; *P* = 0.011) and in the hospital (45.6% vs. 33.5%, respectively; *P* = 0.006) (Table 3). Mortality rates did not differ significantly between younger men and women.

When we compared the duration of mechanical ventilation, ICU mortality and hospital mortality according to the indication for mechanical ventilation, gender, and age, we found that there were no differences in the duration of mechanical ventilation between mechanically ventilated men and women in the overall study population or in both age groups. We found that the ICU mortality and hospital mortality rates for men were significantly higher than those for women postoperatively in the overall study population (11.8% vs. 4.7%, *P* = 0.030 and 14.9% vs. 5.5%, *P* = 0.009, respectively), although there was no significant difference in the APACHE II scores between the men and women (*P* = 0.749). Similarly, this trend was also shown in the older age group (*P* = 0.071 and *P* = 0.033, respectively). No differences in ICU mortality and hospital mortality were found between the men and women after stratification based on other categories and age (see Additional files 1-3).

In the overall study population, as well as in both age groups, men had higher hospital and ICU costs than women. There was no significant difference in the occurrence of withholding or withdrawing life-sustaining treatments between men and women in the overall study population or in either age group. Overall, the proportion of women who were discharged directly home was higher than that of men.

### Multivariate logistic regression analyses

Using multivariate logistic regression analysis, we found that age, gender, the source of ICU admission (medical ward), APACHE II scores, hospital LOS, events occurring over the course of MV, and the decision to withhold or withdraw life-sustaining treatments were independently associated with hospital mortality in the overall study population (Table 4).

**Table 4:** Multivariate logistic regression analysis of factors independently associated with hospital mortality

Variables	All patients (***n*** = 853)		< 65 years (***n*** = 328)		≥ 65 years (***n*** = 525)	
	AOR (95% CI)	***P*** value	AOR (95% CI)	***P*** value	AOR (95% CI)	***P*** value
Age (years)	1.026 (1.014–1.039)	< 0.001	1.013 (0.986–1.040)	0.356	1.062 (1.030–1.096)	< 0.001
Male gender	1.611 (1.101–2.356)	0.014	0.879 (0.446–1.731)	0.709	2.074 (1.315–3.269)	0.002
ICU admission source: Medical ward	1.650 (1.092–2.493)	0.018	2.330 (0.993–5.464)	0.052	1.393 (0.864–2.245)	0.174
APACHE II scores	1.079 (1.055–1.105)	< 0.001	1.080 (1.038–1.124)	< 0.001	1.096 (1.066–1.127)	< 0.001
Hospital LOS (days)	0.984 (0.977–0.992)	< 0.001	0.995 (0.982–1.008)	0.446	0.986 (0.978–0.994)	< 0.001
Events occurring over the course of MV						
Barotrauma ^a^	10.613 (2.942–38.285)	< 0.001	14.561 (2.619–80.957)	0.002	9.075 (1.530–53.820)	0.015
VAP	1.763 (1.103–2.819)	0.018	2.199 (0.916–5.281)	0.078	2.004 (1.182–3.397)	0.010
Reintubation	2.271 (1.028–5.021)	0.043	1.974 (0.396–9.849)	0.407	2.541 (1.052–6.133)	0.038
PMV ^b^	1.869 (1.047–3.335)	0.034	2.543 (0.974–6.640)	0.057	1.493 (0.754–2.956)	0.250
Withhold/withdraw life sustaining treatments	10.928 (6.629–18.016)	< 0.001	17.021 (7.617–38.037)	< 0.001	9.715 (5.212–18.109)	< 0.001

*AOR* adjusted odds ratio, *CI* confidence *interval*, *ICU* intensive care unit, *APACHE II* Acute Physiology and Chronic Health Evaluation II, *LOS* length of stay, *MV* mechanical ventilation, *VAP* ventilator-associated pneumonia, *PMV* prolonged mechanical ventilation

^a^ Barotrauma refers to the development of at least one of the following: interstitial emphysema, pneumothorax, pneumomediastinum, pneumoperitoneum or subcutaneous emphysema

^b^ PMV was defined as the need for mechanical ventilation for more than 21 days

To study whether gender-related differences might depend on age, we prospectively chose a cut-off value of 65 years of age. Therefore, we used separate models for patients younger than 65 years old and for those 65 years or older. We found that male gender was not independently associated with hospital mortality among patients younger than 65 years of age. However, after adjusting for all other factors, we found that male gender was independently associated with hospital mortality among older patients (adjusted odds ratio [AOR] = 2.074; 95% confidence interval [CI] = 1.315 to 3.269; *P* = 0.002), as shown in Table 4. The multiple logistic regression analysis also demonstrated that patient age was independently associated with hospital mortality among elderly patients.

## Discussion

The main finding of this study was that there were gender and age-related differences in the clinical outcomes of mechanically ventilated ICU patients. Specifically, we found that male gender was independently associated with hospital mortality in the overall study population as well as among elderly patients, but not among younger patients.

As reported in other studies [2, 3, 7–11], we found that men account for more than half of the patients (63.2%) receiving MV in the ICU. These findings are even more surprising when we consider the gender distribution of the Chinese urban adult population. For example, in 2010, women accounted for nearly 50% of the Chinese urban adult population, and this proportion increased with age [16]. It is not clear why the proportion of women receiving MV in the ICU is generally lower than that of men, but this may be due to differences in treatment preferences or gender bias. Women were less likely than men to be admitted to an ICU and to receive care and life-supporting treatments such as MV, although the severity of illness was similar in men and women or even higher in women [3, 17–20]. This difference in care may stem from less aggressive treatment preferences by women (or their surrogates) [18, 21, 22]. Furthermore, Sagy et al. [23] reported that both the physician and the patient being female was associated with a decreased ICU admission rate of critically ill patients. Their findings indicated the existence of possible gender bias where the patient and the treating physician both being female diminish the likelihood of restricted health resource use.

Although previous studies have explored the effects of gender on the outcomes of mechanically ventilated ICU patients, the findings across these studies are often inconsistent [7, 10, 13]. Our results are also inconsistent with or even contrary to these previous studies. The possible reasons for this inconsistency are as follows: (I) Age has been shown to be independently associated with mortality in mechanically ventilated ICU patients, and this correlation increases with age [5, 7, 13]. The mean age of the patients included in the three studies was significantly different, and all patients were younger than 65 years old. In our study, the mean age of the patients was 66 years old, and 61.5% of the patients were 65 years or older. (II) Two of the three studies did not further explore the effect of the interaction between age and gender on the outcomes of mechanically ventilated patients. Fowler et al. [3] found that sex- and age-related differences exist among ICU admissions, treatment with specific life-supporting interventions, and short- and long-term outcomes. Mahmood et al. [19] also found that there was a statistically significant interaction between gender and age among critically ill patients. In this study, we also demonstrated that there were gender- and age-related differences in clinical outcomes among mechanically ventilated ICU patients. Male gender was found to be independently associated with hospital mortality among elderly patients but not among younger patients. Therefore, when evaluating the effect of gender on the outcomes of mechanically ventilated patients, we should not ignore the age-related effect. (III) In previous studies, mechanically ventilated patients were either all medical patients [10], mainly medical patients [7], or half medical and half surgical patients [13]. In this study, among the younger age group, more than half of the mechanically ventilated patients came from the surgical ward, while in the older age group, this proportion decreased significantly. Reinikainen et al. [24] found that male gender was independently associated with increased hospital mortality among postoperative patients and among patients aged 75 years or older, but not among medical patients. Women had a higher mortality than men after coronary artery bypass graft surgery and a lower mortality with COPD exacerbation. There was no difference in mortality for acute coronary syndrome, sepsis, or trauma among the critically ill patients [19]. Therefore, referral bias or differences in the composition of ICU admission diagnoses may also contribute to inconsistencies in the study results.

When investigating the reasons for the gender differences in the clinical outcomes of mechanically ventilated ICU patients, in addition to considering age, hormonal status should also be an important factor. In numerous clinical and experimental studies, sex hormones have been shown to affect gender-specific immune responses and organ functions after shock, trauma, and sepsis. Specifically, studies have indicated that female hormones are protective in both immune responses and organ functions, whereas male sex hormones are deleterious [25–28]. In fact, the inflammatory response to infection seems to be exacerbated in males compared with females [29]. The available information indicates that sex hormones play a key role in regulating the immune response and organ function. In addition, there were gender differences in neuroendocrine and endothelial responses in critically ill patients, possibly mediated or regulated by sex hormones [28]. Thus, differences in the hormonal status of critically ill patients may partly explain the gender-related differences in the rate of disease progression and response to treatment in ICU patients. In our study, we found that the duration of MV, ICU LOS and hospital LOS were significantly longer for men than for women both in the overall study population and in the different age subgroups. These results imply that women may be more capable of recovering from critical illness or surgery than men.

In an international multicentre prospective study, Esteban et al. [7] reported that the main conditions independently associated with increased mortality in mechanically ventilated ICU patients not only included the factors present at the start of MV but also factors related to patient management and factors that developed during MV. Esteban et al. [9] also found that factors associated with the highest risk of mortality in patients older than 70 years were the development of complications during the course of mechanical ventilation, such as acute renal failure and shock. In our study, we also found that in the overall study population, the source of ICU admission, APACHE II scores, hospital LOS, events occurring over the course of MV, and the withholding or withdrawing of life-sustaining treatments were also independently associated with hospital mortality. Therefore, three types of variables, including the baseline characteristics of the patients at the beginning of MV, factors related to patient management, and events that occurred during MV, may also have accounted for the differences in the outcomes of mechanically ventilated ICU patients based on age and gender. For example, differences in the process of care or gender-based treatment bias may explain gender-based differences in the outcomes of mechanically ventilated ICU patients [10, 13].

To the best of our knowledge, this is the first multicentre retrospective study in China providing data that indicate gender- and age-related differences in clinical outcomes among mechanically ventilated ICU patients. However, we are also aware of several limitations of our study. First, this study was a retrospective study, and the data were obtained from 2012 to 2013, which may impose temporal limitations on the applicability of this dataset. Second, the 14 ICUs included in our study population were all in Beijing, and these ICUs may not be representative of a random sample of Chinese ICUs. Thus, our research results may not be applicable to other regions or countries. Third, our study did not include other important variables, including nutritional status, the degree of organ dysfunction, and other invasive procedures, which may also account for gender differences in outcomes. Finally, our study only collected the ventilator parameters at the beginning of mechanical ventilation, such as initial tidal volumes, PEEP, etc., but did not record the changes in ventilator parameters over the course of mechanical ventilation. As a result, we were unable to investigate the effects of ventilator modes and settings on clinical outcomes in mechanically ventilated women and men. Therefore, it is necessary to conduct more prospective studies specifically designed to address age and gender differences in outcomes of mechanically ventilated ICU patients.

## Conclusion

In this study, we demonstrated the existence of gender- and age-related differences in clinical outcomes among mechanically ventilated ICU patients. The combination of male gender and advanced age is strongly associated with hospital mortality. Our findings merit consideration when designing future clinical trials involving mechanically ventilated patients.

## Supplementary Information

**Additional file 1: Table S1.** Comparison of the duration of mechanical ventilation (hours) between mechanically ventilated women and men, based on stratification of indication for mechanical ventilation and age.

**Additional file 2: Table S2.** Comparison of ICU mortality between mechanically ventilated women and men, based on stratification of indication for mechanical ventilation and age.

**Additional file 3: Table S3.** Comparison of hospital mortality between mechanically ventilated women and men, based on stratification of indication for mechanical ventilation and age.

## Data Availability

The datasets used and/or analysed in the current study are available from the corresponding author on reasonable request.

## Abbreviations

MV: Mechanical ventilation
ICU: Intensive care unit
NMB: Neuromuscular blocker
LOS: Length of stay
APACHE: Acute Physiology and Chronic Health Evaluation
ARDS: Acute respiratory distress syndrome
PBW: Predicted body weight
BMI: Body mass index
NIMV: Noninvasive mechanical ventilation
COPD: Chronic obstructive pulmonary disease
Pao_2_: Partial pressure of oxygen in arterial blood
F_I_o_2_: Fraction of inspired oxygen
Paco_2_: Partial pressure of carbon dioxide in arterial blood
Spo_2_: Pulse oxygen saturation
VCV: Volume-controlled ventilation
PCV: Pressure-controlled ventilation
PSV: Pressure support ventilation
SIMV: Synchronized intermittent mandatory ventilation
PEEP: Positive end-expiratory pressure
VAP: Ventilator-associated pneumonia
PMV: Prolonged mechanical ventilation
CNY: Chinese Yuan
AOR: Adjusted odds ratio
CI: Confidence interval
