# Supplementary material for: Gender- and age-based differences in outcomes of mechanically ventilated ICU patients: a Chinese multicentre retrospective study
Source: BMC Anesthesiol. 2022 Jan 10;22:18. doi: 10.1186/s12871-021-01555-8 (PMC8744292; doi:10.1186/s12871-021-01555-8)
Supplement: Supplementary file 1 — Additional file 1: Table S1. Comparison of the duration of mechanical ventilation (hours) between mechanically ventilated women and men, based on stratification of indication for mechanical ventilation and age. [file 12871_2021_1555_MOESM1_ESM.docx]

**Table 1** Comparison of the duration of mechanical ventilation (hours) between mechanically ventilated women and men, based on stratification of indication for mechanical ventilation and age

| **Variables** | **All women**  **(n = 314)** | **All men**  **(n = 539)** | ***P***  **value** | **Women < 65 years (n = 120)** | **Men < 65**  **years (n = 208)** | ***P* value** | **Women ≥ 65 years (n = 194)** | **Men ≥ 65**  **years (n = 331)** | ***P* value** |
| --- | --- | --- | --- | --- | --- | --- | --- | --- | --- |
| ARDS | 143 (70, 217) | 162 (96, 322) | 0.204 | 147 (65, 225) | 133 (87, 391) | 0.564 | 136 (70, 206) | 168 (112, 288) | 0.303 |
| APACHE II | 14 (10, 19) | 19 (13, 23) | 0.032 | 12 (8, 17) | 12 (10, 19) | 0.897 | 16 (12, 21) | 20 (18, 24) | 0.036 |
| Postoperative | 56 (38, 112) | 64 (38, 163) | 0.306 | 59 (39, 90) | 69 (38, 167) | 0.154 | 56 (37, 134) | 56 (32, 150) | 0.922 |
| APACHE II | 12 (8, 15) | 11 (8, 16) | 0.749 | 10 (7, 12) | 9 (6, 16) | 0.944 | 13 (10, 18) | 12 (9, 16) | 0.541 |
| Congestive heart failure | 106 (69, 181) | 152 (80, 410) | 0.085 | 68 (38, 151) | 137 (50, 283) | 0.556 | 125 (72, 188) | 221 (80, 428) | 0.213 |
| APACHE II | 18 (15, 25) | 19 (16, 24) | 0.852 | 18 (13, 25) | 23 (9, 29) | 0.730 | 18 (14, 26) | 19 (16, 23) | 0.967 |
| Aspiration | 154 (104, 431) | 165 (71, 374) | 1.000 | - | 112 (92, 556) | - | 154 (104, 431) | 180 (78, 350) | 0.953 |
| APACHE II | 23 (16, 26) | 20 (14, 27) | 0.665 | - | 20 (18, 24) | - | 23 (16, 26) | 21 (13, 26) | 0.599 |
| Pneumonia | 204 (101, 490) | 200 (102, 312) | 0.481 | 120 (79, 240) | 143 (99, 214) | 0.569 | 284 (113, 551) | 216 (102, 419) | 0.323 |
| APACHE II | 19 (14, 24) | 22 (17, 26) | 0.053 | 17 (12, 21) | 19 (14, 24) | 0.363 | 20 (14, 24) | 23 (18, 27) | 0.071 |
| Sepsis | 135 (68, 294) | 163 (112, 296) | 0.421 | 68 (56, 336) | 166 (132, 228) | 0.407 | 174 (87, 280) | 160 (72, 394) | 0.866 |
| APACHE II | 21 (15, 27) | 21 (16, 24) | 0.562 | 20 (15, 24) | 17 (13, 21) | 0.535 | 21 (18, 35) | 21 (16, 26) | 0.735 |

**Table 1** (continued)

**Table 1** (continued)

| **Variables** | **All women**  **(n = 314)** | **All men**  **(n = 539)** | ***P***  **value** | **Women < 65 years (n = 120)** | **Men < 65**  **years (n = 208)** | ***P* value** | **Women ≥ 65 years (n = 194)** | **Men ≥ 65**  **years (n = 331)** | ***P* value** |
| --- | --- | --- | --- | --- | --- | --- | --- | --- | --- |
| Trauma | - | 76 (63, 257) | - | - | 76 (66, 343) | - | - | 84 (56, -) | - |
| APACHE II | - | 12 (5, 21) | - | - | 9 (5, 18) | - | - | 23 (20, -) | - |
| Cardiac arrest | 172 (44, 387) | 326 (139, 411) | 0.264 | 36 (32, 40) | 347 (286, 386) | 0.071 | 218 (114, 401) | 254 (112, 436) | 0.968 |
| APACHE II | 27 (21, 37) | 19 (12, 26) | 0.035 | 19 (13, 25) | 21 (11, 27) | 1.000 | 29 (23, 40) | 19 (13, 25) | 0.026 |
| COPD or asthma | 192 (62, 362) | 214 (111, 437) | 0.247 | 192 (-) | 83 (82, 298) | 1.000 | 199 (59, 362) | 220 (128, 422) | 0.235 |
| APACHE II | 15 (12, 22) | 18 (15, 25) | 0.172 | 19 (-) | 20 (14, 21) | 1.000 | 15 (12, 22) | 18 (15, 25) | 0.119 |
| Other CPDs | 164 (122, 432) | 174 (88, 797) | 0.962 | 130 (109, 287) | 188 (151, 189) | 0.786 | 513 (432, -) | 159 (77, 864) | 0.889 |
| APACHE II | 16 (9, 21) | 18 (13, 24) | 0.475 | 13 (9, 17) | 21 (18, 27) | 0.143 | 19 (16, -) | 16 (9, 21) | 0.667 |
| Coma | 361 (66, 474) | 156 (65, 563) | 0.968 | 96 (37, 368) | 126 (53, 563) | 0.447 | 382 (117, 564) | 173 (145, 732) | 0.697 |
| APACHE II | 23 (18, 31) | 22 (16, 30) | 0.717 | 18 (14, 20) | 19 (15, 31) | 0.661 | 28 (23, 33) | 26 (20, 30) | 0.456 |
| Other | 116 (45, 169) | 110 (67, 271) | 0.586 | 81 (41, 129) | 110 (96, 290) | 0.256 | 169 (65, 344) | 111 (47, 230) | 0.646 |
| APACHE II | 18 (14, 26) | 18 (9, 23) | 0.586 | 16 (13, 19) | 16 (8, 21) | 0.961 | 26 (19, 30) | 21 (11, 25) | 0.328 |

Data are expressed as the median (interquartile range). *ARDS* acute respiratory distress syndrome, *APACHE II* Acute Physiology and Chronic Health Evaluation II, *COPD* chronic obstructive pulmonary disease, *CPD* chronic pulmonary disease
