# Supplementary material for: Gender- and age-based differences in outcomes of mechanically ventilated ICU patients: a Chinese multicentre retrospective study
Source: BMC Anesthesiol. 2022 Jan 10;22:18. doi: 10.1186/s12871-021-01555-8 (PMC8744292; doi:10.1186/s12871-021-01555-8)
Supplement: Supplementary file 3 — Additional file 3: Table S3. Comparison of hospital mortality between mechanically ventilated women and men, based on stratification of indication for mechanical ventilation and age. [file 12871_2021_1555_MOESM3_ESM.docx]

**Table 3** Comparison of hospital mortality between mechanically ventilated women and men, based on stratification of indication for mechanical ventilation and age

| **Variables** | **Women**  **(n = 90)** | **Men**  **(n = 191)** | ***P* value** | **Women < 65 years (n = 25)** | **Men < 65**  **years (n = 40)** | ***P* value** | **Women ≥ 65 years (n = 65)** | **Men ≥ 65**  **years (n = 151)** | ***P* value** |
| --- | --- | --- | --- | --- | --- | --- | --- | --- | --- |
| ARDS | 12 (40.0) | 22 (46.8) | 0.557 | 7 (43.8) | 5 (31.3) | 0.716 | 5 (35.7) | 17 (54.8) | 0.235 |
| APACHE II | 14 (10, 19) | 19 (13, 23) | 0.032 | 12 (8, 17) | 12 (10, 19) | 0.897 | 16 (12, 21) | 20 (18, 24) | 0.036 |
| Postoperative | 7 (5.5) | 29 (14.9) | 0.009 | 2 (3.4) | 11 (11.0) | 0.096 | 5 (7.2) | 18 (18.9) | 0.033 |
| APACHE II | 12 (8, 15) | 11 (8, 16) | 0.749 | 10 (7, 12) | 9 (6, 16) | 0.944 | 13 (10, 18) | 12 (9, 16) | 0.541 |
| Congestive heart failure | 8 (34.8) | 14 (56.0) | 0.141 | 1 (20.0) | 1 (25.0) | 1.000 | 7 (38.9) | 13 (61.9) | 0.205 |
| APACHE II | 18 (15, 25) | 19 (16, 24) | 0.852 | 18 (13, 25) | 23 (9, 29) | 0.730 | 18 (14, 26) | 19 (16, 23) | 0.967 |
| Aspiration | 2 (50.0) | 7 (46.7) | 1.000 | 0 (-) | 1 (33.3) | - | 2 (50.0) | 6 (50.0) | 1.000 |
| APACHE II | 23 (16, 26) | 20 (14, 27) | 0.665 | - | 20 (18, 24) | - | 23 (16, 26) | 21 (13, 26) | 0.599 |
| Pneumonia | 24 (49.0) | 52 (46.8) | 0.803 | 5 (45.5) | 7 (25.0) | 0.262 | 19 (50.0) | 45 (54.2) | 0.666 |
| APACHE II | 19 (14, 24) | 22 (17, 26) | 0.053 | 17 (12, 21) | 19 (14, 24) | 0.363 | 20 (14, 24) | 23 (18, 27) | 0.071 |
| Sepsis | 6 (42.9) | 14 (41.2) | 0.915 | 3 (42.9) | 3 (20.0) | 0.334 | 3 (42.9) | 11 (57.9) | 0.665 |
| APACHE II | 21 (15, 27) | 21 (16, 24) | 0.562 | 20 (15, 24) | 17 (13, 21) | 0.535 | 21 (18, 35) | 21 (16, 26) | 0.735 |

**Table 3** (continued)

**Table 3** (continued)

| **Variables** | **Women**  **(n = 90)** | **Men**  **(n = 191)** | ***P* value** | **Women < 65 years (n = 25)** | **Men < 65**  **years (n = 40)** | ***P* value** | **Women ≥ 65 years (n = 65)** | **Men ≥ 65**  **years (n = 151)** | ***P* value** |
| --- | --- | --- | --- | --- | --- | --- | --- | --- | --- |
| Trauma | 0 (-) | 2 (18.2) | - | 0 (-) | 1 (11.1) | - | 0 (-) | 1 (50.0) | - |
| APACHE II | - | 12 (5, 21) | - | - | 9 (5, 18) | - | - | 23 (20, -) | - |
| Cardiac arrest | 7 (70.0) | 8 (47.1) | 0.424 | 1 (50.0) | 2 (33.3) | 1.000 | 6 (75.0) | 6 (54.5) | 0.633 |
| APACHE II | 27 (21, 37) | 19 (12, 26) | 0.035 | 19 (13, 25) | 21 (11, 27) | 1.000 | 29 (23, 40) | 19 (13, 25) | 0.026 |
| COPD or asthma | 4 (23.5) | 15 (45.5) | 0.130 | 0 (0.0) | 1 (33.3) | 1.000 | 4 (25.0) | 14 (46.7) | 0.152 |
| APACHE II | 15 (12, 22) | 18 (15, 25) | 0.172 | 19 (-) | 20 (14, 21) | 1.000 | 15 (12, 22) | 18 (15, 25) | 0.119 |
| Other CPDs | 6 (85.7) | 7 (70.0) | 0.603 | 4 (80.0) | 2 (66.7) | 1.000 | 2 (100.0) | 5 (71.4) | 1.000 |
| APACHE II | 16 (9, 21) | 18 (13, 24) | 0.475 | 13 (9, 17) | 21 (18, 27) | 0.143 | 19 (16, -) | 16 (12, 21) | 0.667 |
| Coma | 12 (54.5) | 12 (66.7) | 0.436 | 2 (22.2) | 5 (50.0) | 0.350 | 10 (76.9) | 7 (87.5) | 1.000 |
| APACHE II | 23 (18, 31) | 22 (16, 30) | 0.717 | 18 (14, 20) | 19 (15, 31) | 0.661 | 28 (23, 33) | 26 (20, 30) | 0.456 |
| Other | 2 (18.2) | 9 (39.1) | 0.271 | 0 (0.0) | 1 (9.1) | 1.000 | 2 (40.0) | 8 (66.7) | 0.593 |
| APACHE II | 18 (14, 26) | 18 (9, 23) | 0.586 | 16 (13, 19) | 16 (8, 21) | 0.961 | 26 (19, 30) | 21 (11, 25) | 0.328 |

Data are expressed as the median (interquartile range), and number (percentage). *ARDS* acute respiratory distress syndrome, *APACHE II* Acute Physiology and Chronic Health Evaluation II, *COPD* chronic obstructive pulmonary disease, *CPD* chronic pulmonary disease
